# Recent advances in 3D printing of biodegradable metals for orthopaedic applications

**DOI:** 10.1186/s13036-023-00371-7

**Published:** 2023-08-29

**Authors:** Wenqing Liang, Chao Zhou, Hongwei Zhang, Juqin Bai, Bo Jiang, Chanyi Jiang, Wenyi Ming, Hengjian Zhang, Hengguo Long, Xiaogang Huang, Jiayi Zhao

**Affiliations:** 1 Department of Orthopaedics, Zhoushan Hospital of Traditional Chinese Medicine, Zhejiang Chinese Medical University, 355 Xinqiao Road, Dinghai District, Zhoushan, 316000 Zhejiang Province China; 2 Department of Orthopedics, Zhoushan Guanghua Hospital, Zhoushan, 316000 China; 3 Rehabilitation Department, Zhoushan Hospital of Traditional Chinese Medicine, Zhejiang Chinese Medical University, Zhoushan, 316000 China; 4 Department of Orthopedics, Zhoushan Hospital of Traditional Chinese Medicine, Zhejiang Chinese Medical University, Zhoushan, 316000 Zhejiang Province P.R. China

**Keywords:** Biodegradable metals, 3D printing, Bioactive materials, Orthopaedic therapies, Polymer composites

## Abstract

The use of biodegradable polymers for treating bone-related diseases has become a focal point in the field of biomedicine. Recent advancements in material technology have expanded the range of materials suitable for orthopaedic implants. Three-dimensional (3D) printing technology has become prevalent in healthcare, and while organ printing is still in its early stages and faces ethical and technical hurdles, 3D printing is capable of creating 3D structures that are supportive and controllable. The technique has shown promise in fields such as tissue engineering and regenerative medicine, and new innovations in cell and bio-printing and printing materials have expanded its possibilities. In clinical settings, 3D printing of biodegradable metals is mainly used in orthopedics and stomatology. 3D-printed patient-specific osteotomy instruments, orthopedic implants, and dental implants have been approved by the US FDA for clinical use. Metals are often used to provide support for hard tissue and prevent complications. Currently, 70–80% of clinically used implants are made from niobium, tantalum, nitinol, titanium alloys, cobalt-chromium alloys, and stainless steels. However, there has been increasing interest in biodegradable metals such as magnesium, calcium, zinc, and iron, with numerous recent findings. The advantages of 3D printing, such as low manufacturing costs, complex geometry capabilities, and short fabrication periods, have led to widespread adoption in academia and industry. 3D printing of metals with controllable structures represents a cutting-edge technology for developing metallic implants for biomedical applications. This review explores existing biomaterials used in 3D printing-based orthopedics as well as biodegradable metals and their applications in developing metallic medical implants and devices. The challenges and future directions of this technology are also discussed.

## Introduction

 The proportion of individuals aged 65 years and above is currently approaching 10% of the global population, and this figure is projected to increase twofold by the year 2050 [1]. Due to the effects of aging, these elderly individuals are more prone to health issues, including tissue loss and bone fractures. Addressing these complications often necessitates the use of fixation, replacement, or reconstruction procedures. Metals are commonly employed in these procedures due to the mechanical requirements of hard tissue, facilitating patient mobilization and preventing further complications [2, 3]. Conditions such as bone deformities, nonunion or malunion fractures, massive bone loss, tumors, and far-reaching distressing injuries pose significant clinical challenges as traditional surgical procedures have limited effectiveness in treating these disorders [4, 5]. To address these limitations, the concept of bone tissue engineering (BTE) has been introduced [6, 7]. One promising technique in BTE involves the fabrication of three-dimensional (3D) synthetic structures that are tailored to the specific needs of the recipient and can accommodate cellular and protein integration [7].

Currently, approximately 70–80% of the implants employed in clinical settings consist of niobium, tantalum, nitinol, titanium alloys, cobalt-chromium alloys, and stainless steels [8–10]. In recent years, there has been an increasing emphasis on the investigation of biodegradable metals, including calcium, zinc, iron, and magnesium. Notably, several noteworthy findings have emerged in the past few years [11–15]. These metals have the ability to minimize the adverse implications associated with long-lasting implant materials when used as temporary implant materials. This can result in the avoidance of secondary surgeries, thereby expediting the process of tissue regeneration and minimizing further trauma. One notable example is the use of magnesium-based biomaterials, which have garnered significant interest due to their low cytotoxicity, appropriate elastic modulus that aligns with bone tissue, and favorable potential for bone formation [16].

3D printing is a manufacturing technology that combines different elements such as light, computers, electricity, digital control, machinery, and new materials, enabling a revolution in manufacturing across different industries [17, 18]. In recent years, 3D printing has been increasingly adopted in biomedical applications, allowing for human tissue restoration [19]. Its use extends to orthopedic as well as dental implants, cardiovascular systems, and bioartificial livers [20]. Additionally, 3D printing has been utilized in the development of medical electronics and microfluidic devices.

Owing to the mechanical mismatch between metallic implants and bone, there is a risk of stress shielding which could result in bone resorption and implant failure. Therefore, new techniques are necessary to develop biomimetic devices. Traditional powder and metallurgy casting methods cannot create intricate internal architecture and complex external shapes. But with the advent of 3D printing, biodegradable metallic implants with regulated modulus and a porosity that nearly resembles that of natural bone can now be produced [21]. This reduces stress shielding issues. Additionally, 3D printing has low costs, high repeatability, a brief manufacturing phase, and enabling assembly-line productiveness of metallic implants. It is also connected with computer-aided design (CAD), enabling very flexible customized models. To match particular tissue flaws, this technology makes customized therapy possible in ways that have never been done before [22]. In order to design implants that closely mirror both the functional and structural features of natural bone, much effort has been undertaken in this direction.

The swift advancement in 3D printing techniques has given rise to new printing methods that overcome the drawbacks of laser and electron beam printing. Currently, 3D-printed biometals are mainly utilized for creating implants for tissue repair, orthopedics, and dentistry, as well as surgical tools. The development of liquid metals and 3D-printed biodegradable metals will eventually spread to additional biometal applications, such as biodegradable and/or implantable metal biological electronic devices. The uses of biometals, biodegradable metals that are created by traditional 3D printing techniques, and novel implications resulting from developing 3D printing technology are all covered in this article.

## Advancements in three-dimensional printing technology

**Fig. 1:**
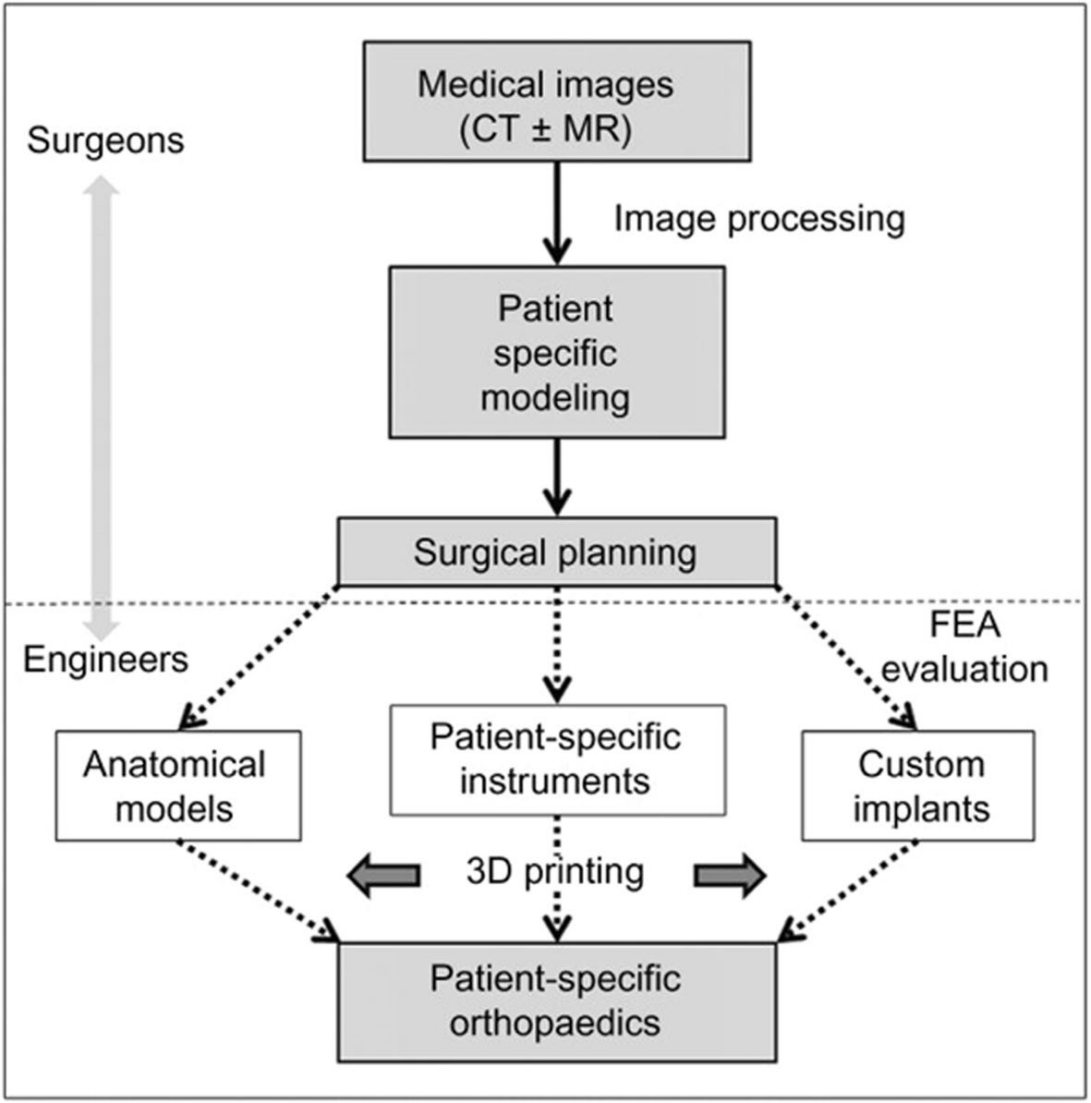
An overview of the clinical workflow involved in the production of patient-specific orthopedic models and implants, starting from the initial stage of image acquisition and concluding with the creation of 3D-printed models and implants [23]

Making actual products from digital models is known as three-dimensional (3D) printing [23]. Computer-aided design (CAD) software can be combined with 3D modeling software, medical scanning methods, or a 3D scanner, to produce a virtual 3D design file. Several 2D cross-section layers are then created using the CAD data. Then, without the need for an intermediary molding phase, a 3D printer creates a 3D structure based on the preset 2D pattern [24]. 3D printing has several advantages, such as design freedom, automation, fast production, negligible leftover generation, customization, and accuracy, [25]. The process of producing a 3D-printed object comprises three critical steps, which are data acquisition, image processing, and 3D printing of the object (see Fig. 1) [24].

### Acquisition of image data: a key step in the process

To accurately characterize the distinct receiver’s anatomy, it is essential to capture the bony structure with precision. Medical imaging modalities such as computed tomography (CT) and magnetic resonance imaging (MRI) are commonly used to obtain 3D medical information with high resolution and accuracy [24, 25]. For orthopedic applications, CT imaging is preferred due to its high contrast and reliable presentation of bone dimensions. However, the requirement of anesthesia for the subject throughout every imaging session and the vast variety of animal species are the two main drawbacks of CT imaging in animals [24]. The medical images acquired by most CT machines are exported in DICOM (digital imaging and communications in medicine), a typical data presentation used to transmit, exchange, and store medical images. As a result, the receiver-specific health imaging records used in orthopedics and 3D printing procedures are connected through DICOM illustrations.

###  Processing of images: enhancing visual data for analysis

The image processing phase necessitates software that can create specific images, known as DICOM, to construct the 3D mesh [24]. This is achieved by transferring the gathered data in DICOM-compliant files to commercial or open-source 3D software programs for 3D object manufacturing. The MPR technique is employed in these programs, which utilize thin axial image slices to produce nonaxial 2D images [23].

Additional reconstructed coronal images are needed during the imaging procedure to correctly portray complex 3D structures for improved clinical visualization and interpretation. When examining skeletal structures and joint alignments in limb abnormalities or fractures, the detailed information on axial elements may not be readily obvious, this technique is very helpful [23]. Another 3D simulation method used to produce 3D mesh representations of the dataset is volume rendering. The DICOM pictures are transformed into 3D representations, which can then be used for CAD file creation or medical diagnostic purposes. Scanner factors including CT reconstruction methods, slice thickness, and radiation magnitude, have an impact on the correctness of 3D renderings [23, 26]. There are several commercial software packages used for medical applications, such as ScanIP (Synopsys, CA, USA), Research Triangle Park, NC, USA), Geomagic Studio (Raindrop Geomagic), Mimics Innovation Suite (Materialise, Leuven, Belgium). In addition, there are open-source applications like Meshlab, Slicer, and InVesalius [24, 27].

#### Segmenting images: partitioning visual data for analysis

The initial step in 3D printing involves segmenting the DICOM representations as well as creating an STL-model. Subsequently, the files of DICOM are imported, and the area of interest, usually the bone, needs to be extracted from the image data. Segmentation is a technique that isolates and extracts the regions of interest by considering specific density and topography information from the image data and removing any unwanted or non-anatomical data. Thresholding is a widely utilized method for separating areas with steady intensity differences from the tissues around them. Following segmentation, the isolated regions can be used to build 3D models [23, 28]. After converting the information to a 3D-CAD suitable format of the file, including the intermediary information in the STL format, primary processing may start. The 3D model quality is strongly correlated with the STL-data quality; hence a high-quality 3D model must be created from high-quality STL data. The STL files may be utilized for 3D printing after both initial and subsequent processing, such as hole correction and noise cancellation. Using CAD software, the profiles of structures are divided into different polygons, often triangles, to generate a 3D mesh model. The resolution of the 3D model is in direct correlation with the number of polygons used, but this can also significantly increase the data size and processing time [23, 28]. To divide DICOM pictures into STL data, utilize medical software like Mimics and 3D Slicer. The final 3D model’s correctness may also depend on scanning factors including slice thickness, radiation strength, and CT reconstructive techniques [23].

#### Processing and examination of data

The DICOM may be simply imported to the image-editing package, where it is transformed into the STL standard 3D format. Before sending the CAD data to the 3D printer to create the item, further modification of the STL files, for instance object geometry correction or triangular mesh optimization is not required but is possible. If there is some need to modify the shape or form of an object, irrespective of how it was constructed, software like Autodesk or freeware for instance BRL-CAD (https:/brlcad.org/) or Openscad (https:/openscad.org/) can be utilized [23, 24, 26].

### Fabrication of objects

The crucial step in producing a 3D model is to create the STL data, and then the STL files can be imported into proprietary applications associated with the commercial software or printer for example Fusion 360 with Netfabb® and KISSlicer, or ReplicatorG. It is crucial to ensure that the final stage package is compatible with the printer that is being used. It is important to utilize “G-code” creation software to create G-code for the purpose of printing the actual 3D model. The STL files used to represent the 3D model are divided into cross-sectional layers using CAD program. The 3D printing method can create the 3D physical model by sequentially adding layers of material. However, each step of the procedure, including STL analysis, 3D printer output, G-code data creation, and DICOM image segmentation, affects how accurate the final 3D model will be [23, 28].

## Applications of metal 3D printing in biomedicine

**Fig. 2:**
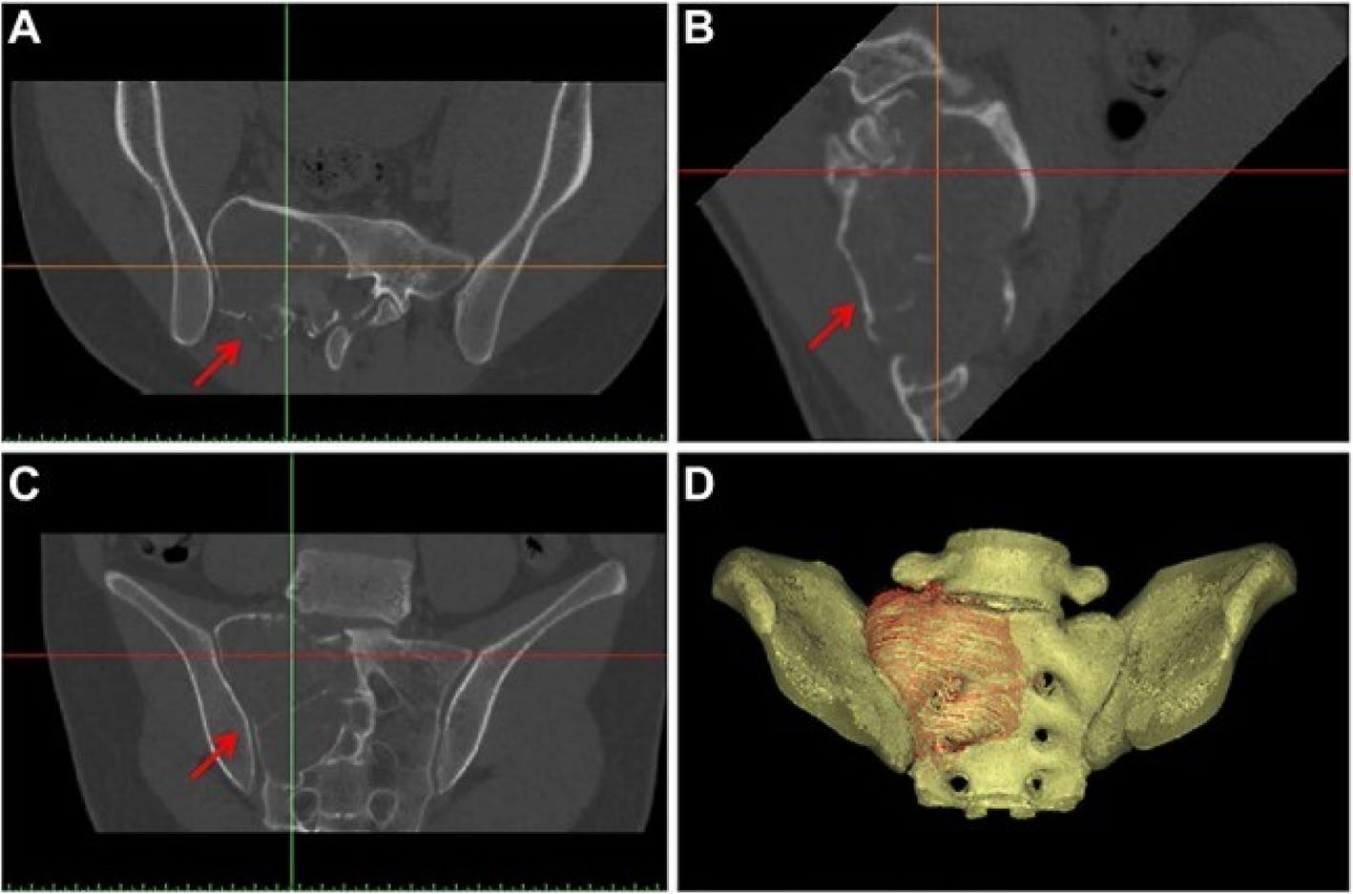
The axial computed tomography (CT) images were obtained from a patient diagnosed with low-grade osteosarcoma that affects the sacrum. The areas of interest are indicated by the red arrows [23]

Techniques for additive manufacturing have made it possible to produce metallic implants with intricate internal structures and custom-made medical implants built for specific individuals, making them very promising for use in clinical settings [29]. One such model is the use of 3D printing to mass-produce highly accurate anatomical models that orthopedic surgeons can use for surgical preparation. Additionally, by creating computer-aided design (CAD) models from patients’ magnetic resonance imaging (MRI) or X-ray computed tomography (CT) images, 3D printing technology may be used to build patient-specific implants. As a result, 3D printing processes are ideal for producing medical equipment made of biometals. The approaches for 3D printing biometals that are currently popular are introduced in this section (Fig. 2**)** [30–34].

###  Laser-based metal sintering techniques

SLS (selective laser sintering) was first put out and patented in 1989 [35]. Liquid-phase sintering is the metallurgical mechanism used in this procedure. Powder densification is accomplished through liquid-phase solidification bonding and solid-phase particle rearrangement. The formation procedure partly melts the powder material while keeping its solid phase core [36]. The two main components of SLS are generally a powder cylinder and a shaping cylinder. The powdering roller evenly distributes the material powder on the forming cylinder’s piston as it rises in the powder cylinder. A computer controls the two-dimensional (2D) scanning path of a laser beam according to the prototype slicing model to selectively sinter the substance of solid powder and generate a layer of the sample. After each layer has been completed, the functioning piston is lowered down one thickness of the layer, the new powder is added, and the new layer is then laser-scanned. Up until the layers are piled to create the required 3D sample, this procedure is repeated. The powder does not totally melt because the semisolid liquid-phase sintering mechanism used in the SLS process prevents it from doing so. Due to the existence of solid-phase particles, the component that develops has high levels of density and porosity even if the stress caused by heat of the forming material is somewhat decreased. This might result in process flaws including poor surface roughness and low tensile strength. Despite this, SLS offers certain benefits, including a broad range of molding options and a straightforward molding procedure (which doesn’t require any support) [37, 38].

### Selective laser melting

SLM (selective laser melting) is a concept that has been around since 1995 [36] and relies on the same ideas as selective laser sintering (SLS). SLM utilizes high-energy fiber lasers and precise powder spreading to entirely melt powder for fast production of metal components [39]. To avoid the metal interacting with other gases that are at high temperatures during the SLM process, the chamber is either inert gas- or vacuum-protected. The utilization of a greater laser energy density and tighter directing point produces functional components with superior dimensional precision and roughness of the surface. SLM may produce operational metal parts without the need for intermediary operations. The parts manufactured by SLM have high densities, strong mechanical properties, and metallurgically bonded structures without requiring post-processing [40]. SLM does not require specially prepared raw materials and may be used using single or multi-component materials. SLM can significantly decrease production time and lower part cost while providing design freedom. SLM lowers material waste and may be used to produce a broad variety of materials. The direct production of functional components with complex geometries is possible with SLM, which makes it the best option for producing singular or low-volume parts.

In recent years, selective laser melting (SLM) has emerged as a major trend in rapid prototyping research [41]. SLM is a versatile technology that is able to print a wide range of materials, such as polymers [42], metals [43], metal-ceramic mixtures [36], and metal-polymer mixtures [44], enabling the creation of near-density metal parts in almost any shape [39]. Due to its ability to produce high-precision, fully metallurgical parts, SLM is frequently used in the medical field to manufacture complex implants with excellent biocompatibility [45], femoral implants [46], and dental restorations [47]. Medical metals, including stainless steel, titanium alloys, nickel-based super alloys, and cobalt-based alloys, are commonly processed with SLM due to its ability to produce highly accurate and surface-finished parts without requiring additional processing steps that traditional procedures (e.g., machining and casting) may necessitate [36]. SLM is also ideal for the fabrication of functional or complex gradient structures with precise proportions, which makes it well-suited for producing metallic scaffolds and implants [48]. SLM 3D printing has thus developed into a potent additive manufacturing approach for the production of unique, complex bio-metallic devices [49].

###  Laser-assisted metal deposition

Since it was initially introduced in the 1990s, laser direct metal deposition (LDMD) has undergone extensive development all around the world [50]. It is also known by different names such as laser rapid forming (LRF), laser-engineered net shaping (LENS), directed metal deposition (DMD), and so on, but they all have the same basic principle. In LDMD, a laser beam concentrates on a spot while a nozzle gathers metal powder on its operation plane. The powder that the laser beams on solidifies, forming a layered entity there [51]. In contrast to SLS/SLM, LDMD uses a nozzle to distribute the metal powder. It has the benefit of producing huge volumes of components and mixing several types of metal powder in the nozzle to produce certain metal alloys. LDMD can be used to manufacture complete parts and for adding, repairing, and coating feature structures. It has the potential to produce materials with pore- or metal-specific gradients, such as shape memory alloys, stainless steels, and titanium alloys, which are more flexible geometrically than materials printed using other methods [52]. For instance, employing LENS for load-bearing bone, Xue et al. [53] developed net form porous titanium [54]. The mechanical parameters of the porous titanium implant were more similar to those of natural bones in comparison to dense titanium, with a Young’s modulus range of 2.6 to 44 GPa and mechanical strength of 24 to 463 MPa. Enhanced cell proliferation and adhesion were seen in in-vitro tests, with an ideal hole size of more than 200 μm. The interconnected pores in the porous Ti6Al4V alloy samples dramatically raised the content of calcium within the implant in male Sprague-Dawley rats for a period of sixteen weeks, showing that biological tissue might develop inside the implant. This study also shown that the overall number of holes in the implant is a significant determinant of tissue ingrowth [55].

LDMD has a precision above 1 mm because of the large laser focusing spot. A dense metal product with metallurgical bonding may be produced, but further machining is needed before it can be used because of its unsatisfactory dimensional precision and surface polish. The deposited material experiences several complicated thermal cycling procedures at various locations using the layer-additive technique used by LDMD, including melting and numerous reheating cycles at lower temperatures. It is challenging to manage the structure and composition required for desired components due to the numerous phase transitions and microstructural changes caused by this thermal behavior. Despite the tiny laser beam quickly creating a molten pool, this causes melting instability and accelerates solidification. The LDMD-shaped pieces may distort or break as a result of the complicated residual stresses produced by the rapid temperature variations that occur during solidification. The two main disadvantages of LDMD technology are the inability to regulate composition and microstructure as well as the production of residual stress [56].

The 3D printing technology that employs high-energy laser sources to melt and fuse metal materials (powder, wire) into layers can produce metallic parts with mechanical properties comparable to those manufactured by traditional methods. However, the fast heating and cooling of the materials caused by the step-by-step scanning and stacking of the electron or laser beams creates a significant temperature gradient and leads to the uneven dispersion of complicated residual stresses. These residual stresses can cause deformation and cracking of metal parts, and negatively affect their mechanical properties and corrosion resistance [30]. There are a number of methods that may be used to lessen or remove these residual tensions [57]: During the design phase, (a) careful consideration should be given to minimizing residual stress; (b) laser scanning mode should be modified by revolving the scanning vector to one processing layer to the following to prevent concentration of pressure on the similar path; (c) uninterrupted sintering and broad area should be avoided; and (d) the powder bed should be heated before the part is annealed to remove stress.

###  Focused electron beam melting

Using high-energy electron beams, SEBM rapidly manufactures 3D samples by heating and melting metal particles in precise patterns. To form a 3D part, a layer of powder is first spread on a surface, followed by selective heating of the metal powder according to the 3D CAD file information. A magnetically guided electron beam helps the molten powder form layer-by-layer connections with the component below. The extra powder is then removed to produce the required 3D sample [58]. The impacts of process variables such as scanning mode, acceleration voltage, powder thickness, action time, focusing current, and electron beam current are now the subject of research [59]. In SEBM, the metal powder is melted using a high-energy electron beam, and the beam is controlled by a magnetic deflection coil without the need for mechanical inertia. During melting or sintering, the metal powder cannot oxidize due to the vacuum atmosphere. The electron beam has several advantages over lasers, such as maintenance costs, better stability, low operational, outstanding material absorption, and great energy utilization. Comparing other techniques, SEBM has lesser part distortion, higher efficiency, denser microstructure formation, and does not require molding support. A magnetic field is used to regulate the focus and deflection length of the electron beam, making it quicker and more sensitive. This is done by adjusting the electrical signal strength as well as direction. High dimensional precision and intricate design for the molded object are difficult to accomplish, nevertheless, since electron beams are unable to focus on a small location [60].

In biometallic devices like acetabular cups, intramedullary rods, and femoral knee implants with exterior porous mesh structure areas, SEBM is a common 3D printing technique [61]. However, owing to SEBM characteristics such component orientation, beam flow, and powder size which provide a difficulty for applying this approach in orthopedic implants, it is challenging to optimize the surface finishing of implants [62].

The printing procedure can be quite costly due to the high expenses associated with metal devices and powder, which can amount to lots of dollars. As the demand for metal 3D printing in manufacturing continues to rise, more resources and scientific research are being allocated to improve the technology and make it more cost-effective. Several new methods are being developed that hold significant potential to reduce costs and encourage new notions for manufacturing, although they are not yet available for commercial use.

###  Laser-based Forward transfer

Metal printing is often restricted to substances with lesser melting points, making the 3D printing of metals including copper or gold challenging or expensive. To solve this problem, a technique known as LIFT was recently created [63]. A thin covering of metal material known as a “donor film” is used in LIFT, a direct printing technique that differs from traditional metal 3D printing processes. A liquid droplet is ejected onto a transparent substrate known as the “receiver” when a pulsed laser targets it on the donor film, causing a thermal pressure wave or evaporation [64]. This technique has been applied successfully to print metals such as chromium, gold, titanium, nickel, and aluminum [65, 66]. However, thick metal printing by LIFT faces challenges in achieving good adhesion between stacked droplets, resulting in low aspect ratio pillars, sphere-like shapes, and reduced contact surface between subsequent layers. Due to the decreased layer-by-layer deposition accuracy, it is difficult to create massive 3D constructions and overhang [67]. Sacrificial support materials may be required to overcome these limitations. LIFT has been used to fabricate biosensors, and recent advancements have demonstrated the ability to print objects of small-scale with an alloy of silver/copper [68]. The lateral dimensions of the reported range of LIFT in the micrometer scale, with the smallest size being nearly 3 μm [69]. Copper and gold were used as the primary building blocks in recent tests to print a 3D tower that was 2 mm high and 5 μm in circumference. Low-energy lasers were also used to create disc-like droplets, which improved layers stacking [70].

###  Additive manufacturing via atomic diffusion

A new 3D printing method called ADAM has recently been developed for layer-by-layer printing of metal-powders encased in plastic binders [71]. After printing, the plastic binder is detached in a sintering-furnace, leaving behind high-density (95–99%) metal powder. The issue of poor interlayer strength seen in previous 3D printing procedures is effectively resolved by the simultaneous sintering of the whole component, which encourages the formation of metal crystals via the adhesive layer. In addition, the ADAM printer is capable of producing geometric proportions that are not possible with conventional techniques of metal 3D printing. Its maximal printing size is 250 millimeters by 220 millimeters by 200 millimeters. Additionally, it provides cloud-based online process laser detection that enables customers to keep an eye on each printing layer. The printer has a remote cloud-based access metal material handling system. The printer can 3D-print 303 and 17 − 4 stainless steel, however, its compatibility with A-2, Ti6Al4V, and D-2 steels is currently being examined. The advantages of ADAM over conventional metal 3D printing methods are numerous. It generates high-quality part surfaces requiring post-treatment, builds accurate and complicated structures, has good isotropic performance, is 100 times faster than conventional machining, costs one-tenth as much as conventional metal 3D printing, and is best suited for batch production.

###  Nanoparticle-based jetting

A new metal 3D printing technique called nanoparticle jetting (NPJ) was released in 2016. It differs from traditional metal 3D printing in that it uses liquid ink to enclose metal powder particles instead of using metal powder particles [72]. Large metal pieces are broken down into nanoparticles as well as embedded in a binder to make the ink, which results in consistent ink [72]. The ink’s metal particles are disseminated and suspended in the ink, which is then expelled via a nozzle to produce a layer-by-layer print. This ink-based approach smooths the entire product, which is beneficial. The printing process uses a standard inkjet printhead as the deposition tool. Following the printing process, any surplus binder in the chamber will evaporate as a result of the heating, and the metal component will be all that is left. The precision of this process is around 1 micrometer (m), and the forming temperature is approximately 300 °C. Because it is able to drop 221 million droplets of ink each second, the NPJ printing process is five times quicker than standard laser printing. The use of NPJ results in cost savings, reduced material waste, and the ability to make virtually any complicated form. The components have a high level of accuracy and surface quality, and the working processes have been made easier and safer. However, its temperature tolerance is lower compared to conventional metal 3D printing, which remains its main disadvantage. Overall, NPJ is a simple and clean method that does not require designing and removing composite support structures [71].

###  Binder jetting technology/inkjet 3D-printing

Governed by a CAD file, a precise water jet is printed onto a metallic powder bed in layers by an ink cartridge [73]. The required mechanical strength is subsequently achieved by sintering the printed metal components in a furnace. This method is unable to use a laser or an electron beam for melting the metallic particles, in contrast to other 3D metal printing processes. The inexpensive cost of the equipment and the thermally regulated sintering process are the key benefits of inkjet 3DP. Even though Inkjet 3DP is less precise than SLM, SEBM, and LENS, its ability to fabricate bio-metallic devices quickly and at low cost makes it appropriate for biomedical applications [74].

###  Extrusion-based 3D printing

Extrusion-based 3D printing has gained prominence as a promising printing technique due to its widespread acceptance, user-friendly nature, ability to accurately print intricate geometries through CAD, and utilization of various solidification techniques. However, it should be noted that this method requires the use of materials possessing specific printability qualities [75, 76]. The adoption of this printing method is comparatively more cost-effective and potentially less challenging as compared to the aforementioned alternative printing methods. Furthermore, the fabrication and geometrical parameters can be readily adjusted in order to meet the scaffold specifications of the user, such as achieving a high modulus and ensuring structural integrity, among others. An example of this is the utilization of cylindrical fibers in layer-by-layer fabrication, which offers enhanced structural integrity in comparison to alternative 3D printing methods such as droplet or inkjet-based fabrication. The complexity of tissue replication or structural support necessitates the utilization of multiple materials, leading to the need for multi-material extrusion [77, 78]. Multi-component systems have the capacity to readily generate interfacial tissues in various biological structures such as organogenesis, vasculature, muscle, and bone.

**Fig. 3:**
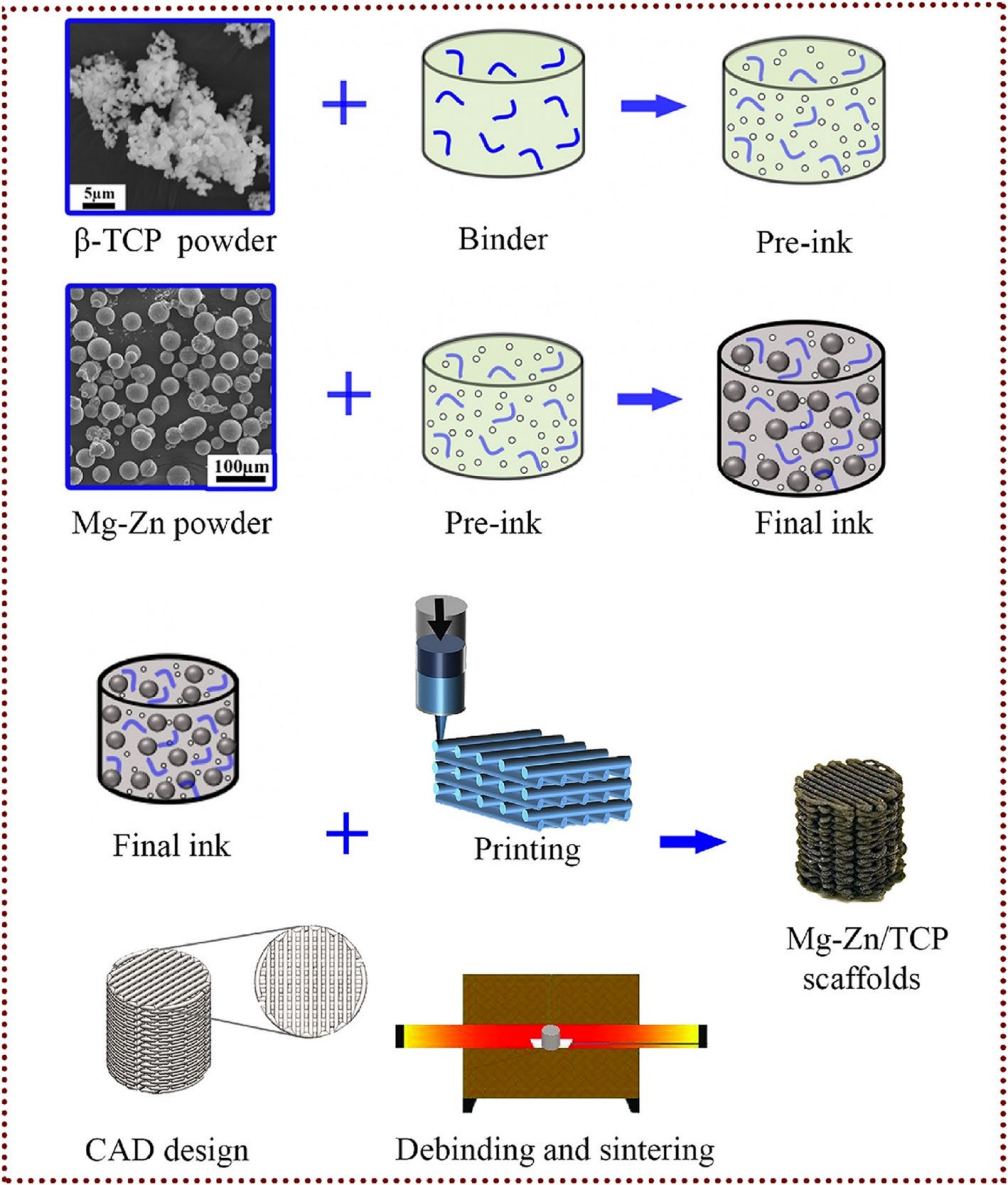
A step-by-step diagram showing how composite scaffolds are made using extrusion-based 3D printing [80]

The use of extrusion-based 3D printing, coupled with subsequent debinding and sintering processes, presents a robust methodology for the production of porous scaffolds. This technique proves particularly advantageous in cases where the materials involved pose significant difficulties when subjected to alternative additive manufacturing methods. The fabrication of porous iron scaffolds featuring a lay-down pattern was effectively achieved through the use of extrusion-based 3D printing. These scaffolds possess improved biodegradability and exhibit mechanical properties that closely resemble those of natural bone. Consequently, they hold great promise for utilization as bone substitutes [78]. Putra et al. employed extrusion-based 3D printing methodologies to manufacture biodegradable Fe-Mg scaffolds. These scaffolds were characterized by non-ferromagnetic properties and demonstrated improved rates of biodegradation [79]. These scaffolds effectively resolved the primary constraints associated with biodegradable scaffolds, such as their low biodegradability and lack of compatibility with magnetic resonance imaging. Dong et al. recently reported an extrusion-based additive manufacturing methodology for the production of biodegradable Mg-Zn/bioceramic composite scaffolds. These scaffolds hold promise for addressing femoral nonunion accompanied by significant segmental bone defects (Fig. 3)﻿ [80]. The biodegradation kinetics of Mg-matrix composites were modulated by the incorporation of bioceramic particles into the Mg-matrix. The Mg-matrix composites demonstrated enhanced cytocompatibility and mechanical properties in comparison to conventional magnesium alloys.

## Suitable materials for 3D printing

Currently, many investigations are underway to create fresh biomaterials suitable for 3D printing [25]. The term “biomaterials” describes compounds of organic or synthetic origin that are integrated with tissue from life to sustain, replace, or regenerate organs or tissues. The biomaterial which is employed for 3D printing is affected by the final object’s intended usage [81]. For instance, a biological material used in orthopedic 3D printing has to be efficiently printable, have high biocompatibility, be subject to controlled biodegradation, have useable mechanical properties, and possess a designed effectively architecture [81, 82]. The substance must be sterilizable for surgical uses [23]. Despite the fact that 3D printing has been effective in a number of medical applications, there are now only a few materials that can be used for 3D printing. In 3D printing for orthopedic applications, the most commonly used biocompatible and implantable materials are ultra-high-molecular-weight polyethylene (UHMWPE), polyetheretherketone (PEEK), ceramics, stainless-steel (SS) alloy, cobalt–chrome (CoCr) alloy, and Titanium (Ti6Al4V) alloy, [25]. Biomaterials used in 3D printing may be loosely categorized into four types based on their chemical makeup [81].

### Advanced ceramic and glass materials

Due to their possible osteoinductive and osteoconductive qualities, high stiffness, and resemblance to the mineral phase of bones, bioceramics have recently been used for building 3D-printed implants [4, 83]. Calcium hydroxyapatites (HA), a component of calcium-phosphate ceramics (CaP ceramics), are artificial materials with a structure resembling the natural bone matrix. Common CaP ceramics utilized in bone repair include HA, TCP, and BCP [84]. In the first two weeks following implantation, bioactive glasses, also known as bioglasses, are ceramics composed of synthetic silicates that are swiftly resorbed, allowing for speedy bone development and implant ingrowth. In spite of the fact that bioactive glasses and CaP ceramics are utilized in the production of 3D-printed scaffolds, the materials’ restricted mechanical qualities, such as poor fracture-toughness as well as tensile strength, prevent these materials from being used in load-bearing applications. Even though bioactive glasses and to get around this drawback, bioceramics are combined with polymers such as PCL, poly (D, L-lactic acid-co-glycolic acid), or cellulose, they are mixed with specialized reinforcing materials such as carbon nanotubes, graphene, polyethylene, Al2O3, and TiO2 to produce ceramic composites with increased mechanical strengths [83]. Because of their high biocompatibility and osteogenesis qualities [85], graphene and its derivatives have the potential to make significant contributions to the field of material research. The biological and osteogenic features of graphene-based scaffolds populated in vitro with canine adipose-derived mesenchymal stem cells (cAD-MSCs) were investigated by a research team. According to the findings, carbon may enhance cADMSC cell adhesion, proliferation, and osteogenic differentiation and the graphene-based scaffold is extremely biocompatible. In the field of veterinary medicine, novel materials for bone tissue engineering have been suggested, and one such material is scaffolds based on graphene [4]. Ceramics are also utilized to improve osteo-integration between bone tissues and implants and to limit the micromotion that occurs between implants and bone. This is done by reducing the amount of micromotion that occurs. Ceramic femoral heads are not yet utilized in the production of veterinary hip implants; nonetheless, femoral head coating through diamond-like carbon was utilized in the production of the most recent generation of the Zurich cementless hip [86].

### Advanced polymer materials for 3D printing

In the production of 3D-printed bone substitutes, the use of polymers is a common practice because of their potential application as filaments for fused deposition modeling (FDM), solutions for stereolithography apparatus (SLA), powder beads for selective laser sintering (SLS), and gels for direct ink writing (DIW) [87]. This is because polymers can be used in any one of these capacities. PDL, PLA, PGA, or their copolymers, as well as PLGA are the biodegradable polymers that may be utilized in the 3D printing process [87]. 3D printing frequently makes use of PCL, which is not only an FDA-approved biodegradable polymer but also a polymer that can be broken down naturally. Because of its high level of biocompatibility, low rate of degradation, and satisfactory mechanical qualities, PCL is the material of choice for the production of 3D-printed bone scaffolds [5, 88]. The thermoplastic polymer known as high-density polyethylene, or HDPE for short, is a material that finds widespread application in biomedical engineering since it possesses favorable mechanical and thermal characteristics. Polyethylene is used as bearing surfaces for veterinary total hip prostheses [88]. UHMWPE is the type of polyethylene that is utilized in veterinary joint replacements the most, as well as the type that was initially employed [86]. Significant shifts have occurred in the selection of materials used in human THR surgery [88], including a move away from HDPE and toward UHMWPE. In addition, receiver-specific surgical guides, equipment, and prosthesis may be fabricated out of thermoplastic polymers like PLA and acrylonitrile butadiene styrene (ABS). These thermoplastic polymers can be utilized to create 3D models of limbs as well. In addition, polyamides are well-known for the stability, stiffness, flexibility, and shock-resistance qualities that they possess. Recent research has demonstrated that polyamides and HA, when coupled, may be utilized to make porous scaffolds for bone regeneration [89]. These scaffolds have significant load-bearing capabilities and can be used to build porous scaffolds.

Due to its outstanding elasticity, tunable mechanics, biodegradability, and biocompatibility, 3D-printed hydrogel scaffolds display a substantial amount of potential in the creation of tailored scaffolds for BTE at present. Hydrogel and bioinks are generally printed through laser-assisted bioprinting, inkjet, and extrusion 3D printing methods. Due to its outstanding elasticity, tunable mechanics, biodegradability, and biocompatibility, 3D-printed hydrogel scaffolds display a substantial amount of potential in the creation of tailored scaffolds for BTE today. Earlier research has indicated that the cell viability from extrusion-based bioprinting can reach 98% [90]. Dynamic structure hydrogels may enhance the healing efficiency of polymers at the molecular level to minimize cell damage in the course of extrusion printing. The degree to which such structures can mend themselves or the number of times in a row that they are able to do so are the two characteristics that define their level of self-repair performance. Direct ink writing, often known as DIW, is widely regarded as the technique that is employed the most frequently to develop self-healing intelligent frameworks [91].

###  Advanced composite materials for 3D printing

Composites are a type of artificial material created by combining two or more elements with different physical characteristics to achieve synergistic properties. The diverse structure of composites allows them to maintain proper mechanical properties while being highly biocompatible, which makes them useful for 3D printing bone replacements [83]. Some composite materials that have been investigated for this purpose include PCL/PLGA/TCP, PLGA/TCP/HA, and PCL/TCP. For instance, PCL has been combined with beta-TCP, a compound that, in addition to becoming more osteoconductive and biodegradable, is also capable of releasing calcium and promoting bone formation. PCL/-TCP has a stronger capacity to induce bone formation, stimulate the regeneration of bone, and substitute bone than PCL on its own [92]. For the purpose of promoting osteogenesis in a rabbit model, PLA/n-HA composite scaffolds with varied percentages of n-HA were utilized. The results revealed that the PLA/15% n-HA composite scaffold retained its biological activity as well as suitable mechanical qualities in the rabbit model defect [93]. The low elastic modulus of polymers makes it appear as though it might be possible to create prosthetic devices solely from them; nevertheless, their poor strength makes this approach inappropriate. It is not uncommon for metallic prosthetic limbs to fall short of surface compatibility criteria. Because of this, a significant number of contemporary limb prosthesis are fabricated from polymer-based composites, that have great strength-to-weight ratios and are highly biocompatible [87].

## Biomedical metal fabrication using 3D printing

3D printing technology has the potential to revolutionize the medical industry by offering faster, more cost-effective, and more efficient solutions for medical treatments. This technology, combined with imaging and scanning methods such as ultrasonic tests, CT, and MRI, enables mass production and customized fabrication of medical devices. As a result, the applications of 3D printing technology in the medical field are constantly expanding. In fact, some 3D-printed medical devices have already been granted market licenses by the US FDA, indicating the growing acceptance and adoption of this technology in the medical industry.

This section outlines various practical uses of biometals made possible through 3D printing technology. Additionally, current studies on the creation of novel biodegradable metals using 3D printing techniques are also discussed.

###  Titanium-based alloys

Since the 1970s, titanium and titanium alloys have been extensively utilized in the field of biomedical implant materials. This is primarily attributed to their exceptional resistance to corrosion, biocompatibility, and high strength-to-weight ratio [94, 95]. Titanium alloys possess exceptional resistance to corrosion, rendering them highly suitable for various biomedical applications. These applications encompass a range of uses in the field of biomedicine, such as bone plates and screws, orthopedic and dental implants, synthetic joints, heart valve prostheses, pacemakers, and cornea backplates [96]. Ti-64 and CP-Ti are currently regarded as the most preferred metals for use in medical settings. These metals are employed in approximately 90% of all conventional orthopedic implants.

In comparison to bone tissue, dense metal implants exhibit higher density as well as greater stiffness and elastic modulus of elasticity [97]. The utilization of implants may potentially lead to the occurrence of stress shielding. The utilization of 3D printing technology has the potential to produce biodegradable implants that possess a porous structure, which assists in the reduction of stress shielding effects commonly associated with implants. The elastic modulus of a metal prosthesis can be altered by utilizing 3D printing techniques to fabricate porous titanium alloy implants that possess differentiated pores, varying gradient apertures, and three-dimensional penetration within the pores [98]. The utilization of 3D printing technology enables the fabrication of customized titanium alloy implants that can effectively conform to an individual’s anatomical structure. This is especially advantageous due to the corrosion resistance, exceptional biocompatibility, and enhanced strength-to-weight ratios exhibited by titanium and its alloys, rendering them suitable for various biomedical purposes [99]. Consequently, there is a growing expectation regarding the forthcoming surge in demand for 3D printing employing titanium. Currently, the most commonly employed 3D printing processes for titanium alloys are SLM (Selective Laser Melting) and EBM (Electron Beam Melting) [72]. Clinical histology experiments have demonstrated that the osseointegration rate of traditional implants is comparatively slower when compared to 3D-printed implants [100, 101].

**Fig. 4:**
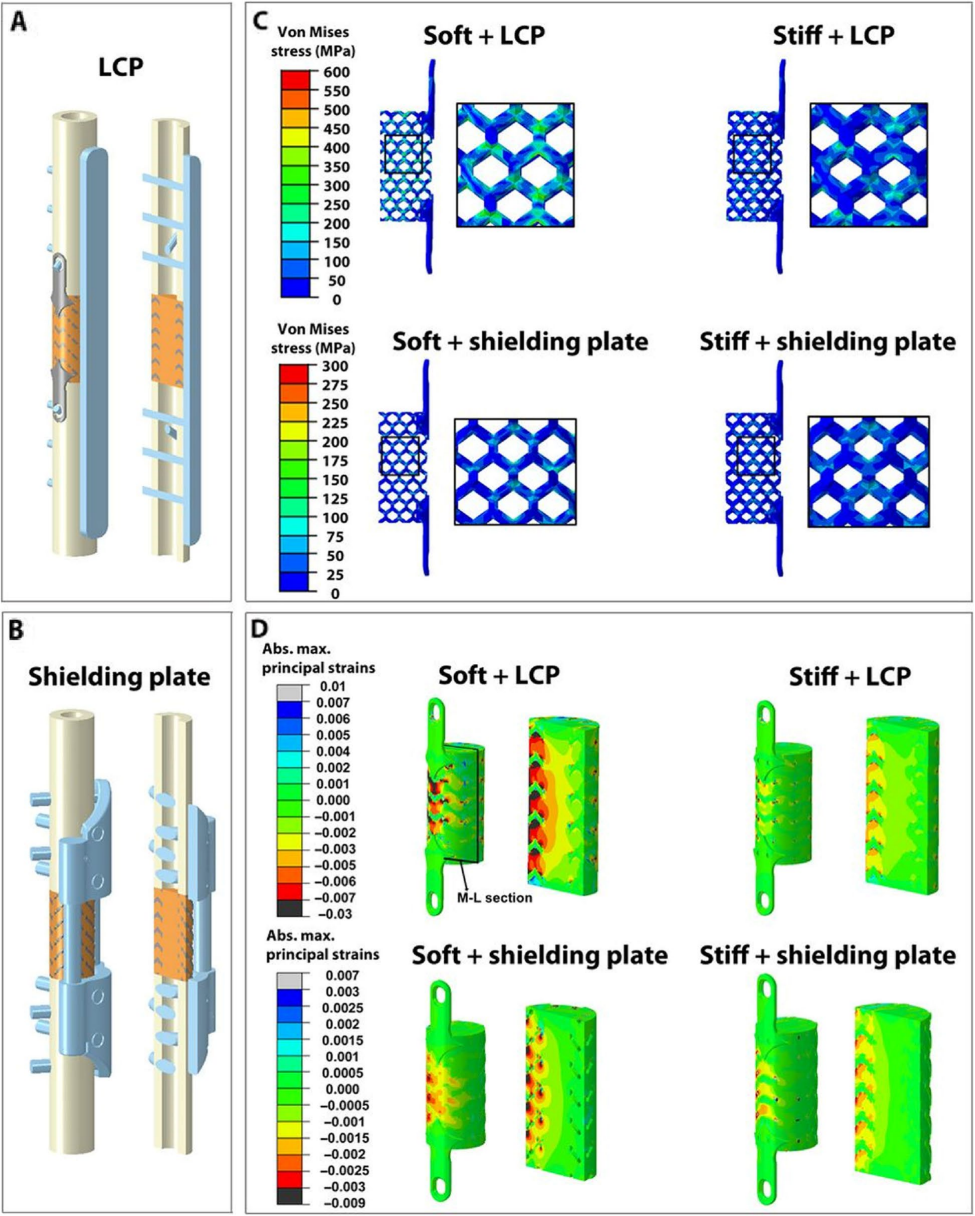
**A** A 3D-finite element (FE) model was constructed to simulate a 4 cm tibia defect in sheep. The model incorporated the use of a locking compression plate (LCP) for stabilization, along with a Ti-mesh scaffold for augmentation. **B** A 3D-FE model was constructed to simulate a 4 cm tibia defect in sheep. The model incorporated a custom-designed shielding plate, made of half-shell steel, to provide stabilization. Additionally, a titanium mesh scaffold was utilized to augment the defect. **C** Honeycomb scaffolds are constructed using titanium struts of uniform length (7 mm) but varying diameters (1.6 and 1.2 mm). The scaffolds are comprised of a cylindrical structure (4 cm in length and 2 cm in diameter) accompanied by a hole that traverses the medullary canal with a diameter of 1 cm. **D** The maximum principal strains observed in the stiff and soft scaffolds were stabilized using LCP at top. The maximum principal strains observed in the stiff and soft scaffolds were stabilized using a shielding plate at the bottom [106]

The SLM molding process was examined by Hollander et al. [102] using Ti6Al4V powder. They discovered that a titanium alloy vertebra formed using SLM had acceptable biocompatibility, which makes it ideal for substituting biological components. Various studies have also shown that the biocompatibility of implants of titanium alloy fabricated using EBM or SLM is acceptable [103, 104]. For instance, Palmquist et al. [105] assessed the osseo-integration of solid Ti6Al4V implants and EBM-printed porous, in disk and cylindrical forms. These implants were placed bilaterally in the subcutaneous of the dorsum in the femur of sheep. The research showed both the solid and porous implants were properly osseointegrated after a 26-week implantation period. The porous implants had an elevated contact rate of bone of up to 57%, which was the highest among the porous implants. In addition, Pobloth et al. produced two mechanically distinct titanium-mesh scaffolds having honeycomb-like patterns by 3D printing using a laser sintering method (Fig. 4) [106]. They subsequently implanted these scaffolds into sheep tibia in order to evaluate the influence that they had on the endogenous bone defect healing. In addition to the findings of this research, several successful operations with 3D-printed implants made of titanium have been carried out in recent years. According to these statistics, tailored medical implants reduce the amount of time spent in hospitals and during surgical procedures, which in turn lowers overall medical expenditures. For instance, in 2011, the first therapeutic transplant procedure was conducted utilizing a client-specific jaw implant manufactured by a 3D printer for a female patient who was 83 years old [107]. This operation was performed on a patient who was also the first person to use a 3D printer. The lower jaw of patient was suffering from chronic bone infection, and given her age, reconstructive surgery was considered a risky procedure. However, using 3D printing processes made it possible to create a titanium jaw implant in just one day, with less weight and a quicker surgical installation time than conventional mandible implants.

###  Tantalum-based alloys

Since the 1940s, Tantalum (Ta) has been a popular choice for the applications of orthopedic and dental, such as vascular clips, cranial-defect repair, nerve repair, and bone markers, due to its good chemical stability and excellent biocompatibility [108]. However, in the past, the use of Ta was restricted due to its high production cost and the complexity associated with the fabrication of modular implants utilizing Ta [109]. Today, porous tantalum implants have been produced for osteogenesis applications, such as bone graft replacements, and surgery has made extensive use of tantalum-based knee, hip, and spinal prostheses [110]. Clinical reports on porous tantalum implants, such as spinal implants, tibial, and acetabular cups, have demonstrated their usefulness in a variety of clinical settings [111]. New manufacturing processes including LENS, SLM, and spark plasma sintering have been utilized to produce porous and solid tantalum components [112]. For instance, Balla et al. [109] effectively coated Ta on Ti via LENS, and the osseointegration characteristics of the Ti surface were greatly enhanced as a result of the Ta coating. They also generated Ta porous materials using LENS with varied porosity and Young’s modulus by altering the porosity from 27 to 55%. These structures ranged from having a porosity of 27% to having a porosity of 55%.

In 2017, a male patient who was 84 years old underwent the first successful operation to repair a severely defective knee joint utilizing an artificial joint cushion of 3D-printed tantalum. The procedure was performed on a patient in the United States. This was a significant achievement as it was the first-time tantalum had been used in a clinical implant. The shape, density, and defects of the patient’s knee joint were analyzed using a 3D CT scan, and a personalized Ta cushion was made up using 3D printing. Because the geometry of bone abnormalities might vary from patient to patient, standardized metal pads are not always capable of correctly reconstructing bone structures for every single patient. Titanium is now the material of choice for the majority of 3D-printed joint pads; however, tantalum is superior in terms of biocompatibility and supports improved bone ingrowth, which makes it a better option for implants. Nevertheless, one significant issue with Ta is its high melting point, which makes it challenging to work with using most 3D printing equipment. Using SLM, a customized 3D-printed tantalum pad was created in order to solve this issue [109].

###  Alloys of cobalt and chromium

The elements cobalt and chromium are the primary constituents of the super alloys known as cobalt-chromium alloys. These alloys have remarkable mechanical qualities in addition to high resistance to corrosion [113]. Because of their high strength, high-heat resistance, more effective durability against wear, and biocompatibility, Co-Cr alloys are frequently employed as load-bearing implants [114]. Although they were first developed for use in artificial joints, dentists and oral surgeons today often employ them in a variety of other applications [115]. The use of 3D-printed Co-Cr alloy porcelain teeth is the top option for non-precious metal porcelain devices [116]. This is because these teeth do not include potentially toxic components such as nickel and antimony.

Nevertheless, even though CoCr alloys are considered ideal for load-bearing implants, they may encounter problems with wear and corrosion when implanted into the human body. This can lead to a loss of implant material and the release of metal ions, which may result in various medical complications. To address this issue, Sahasrabudhe et al. [117] discovered that adding CaP to alloys of CoCrMo can decrease the wear rate. They manufactured CoCrMo alloys containing 3% CaP by using LENS 3D printing, and they discovered the sample wear rate was only one-third of what it would have been if they had used pure CoCrMo alloys instead. In addition, the Co^2+^ and Cr^2+^ release was decreased to a level that is four times lower than that of pure CoCrMo alloys that do not contain CaP.

###  Partially degradable implant material

Metallic materials, including cobalt-based alloys, stainless steels, and Ti alloys, possess several advantageous properties for bone implants. These properties include favorable machinability, resistance to fatigue, and high fracture toughness [118]. However, it should be taken into account that these materials are not biodegradable within the human body and possess Young’s moduli that exceed those of human bone. The phenomenon described can potentially lead to stress shielding, resulting in fractures, loosening, and bone atrophy [119]. In order to tackle this matter, it is possible to manipulate or introduce porosity to materials, thereby facilitating a durable and consistent fixation over an extended period. This is achieved by enabling bone growth into the pores and establishing a mechanical connection between the implant and the bone [120]. Biodegradable metals, including alloys based on magnesium, calcium, iron, and zinc, possess bioactive properties and can undergo complete degradation within the human body. These metallic implant materials provide both biological functionality and mechanical support. These materials possess low Young’s moduli, comparable to bone, rendering them advantageous in mitigating the adverse effects of stress shielding. One potential approach to integrating the beneficial properties of nondegradable and degradable implant materials is through the development of composites comprising these elements. For example, Ti has been identified as a potentially suitable reinforcement phase for magnesium in biomedical applications due to two main reasons. Firstly, Ti and its alloys demonstrate high specific strength, indicating a favorable strength-to-density ratio [121]. Additionally, Ti exhibits good biocompatibility and corrosion resistance, making it a desirable choice among nondegradable implant materials. Secondly, even at elevated temperatures surpassing the melting point of Mg, Ti does not readily form solid solutions or intermetallic compounds when in contact with Mg [122].

In the setting of orthopaedic implant applications, a partially degradable Mg-Ti composite with enhanced compression properties has been successfully developed through the use of ink jet 3D printing and capillary-mediated pressure-less infiltration techniques [123]. The composite material exhibits a low modulus and a high ultimate compressive strength, which are comparable to those of human cortical bone. The corrosion rates for porous Ti are considered to be insignificant, while for Mg-Ti composites, they are less than 1 mm per year. The composite material demonstrated a significant enhancement in the proliferation rate of SAOS-2 osteoblastic bone cells, accompanied by a minimal level of cytotoxicity. Dou et al. developed an Mg-Ti composite through the process of pressure-less infiltration, where pure Mg melt was infused into a 3D printed Ti-scaffold [124]. The composite’s topologically bi-continuous architecture offers higher strengths and lower Young’s modulus compared to dense Ti. The potential degradation of the Mg phase has the capacity to stimulate the formation of bone tissue, thereby facilitating the establishment of a mechanical interlocking mechanism between the Ti-scaffold and the bone. Despite the occurrence of accelerated corrosion, the composite material continues to exhibit non-cytotoxic properties and does not elicit any adverse reactions following its implantation. Liu et al. have successfully developed Mg-composites that exhibit structures resembling fish scales, specifically the double-Bouligand and orthogonal plywood architectures [125]. The fabrication process involved the pressure-less infiltration of a Mg melt into Ti-fibres. The composites exhibit improved mechanical strength and work-hardening capabilities, owing to the presence of a double-Bouligand architecture that effectively redirects crack propagation and enables the Ti-fibres to adaptively reorient. Zhang et al. reported the synthesize of Mg-Ti composites through the process of pressure-less infiltration, wherein a pure Mg melt was introduced into 3D-printed Ti-6Al-4 V scaffolds [126]. The final product yielded composite materials that featured a continuous arrangement of constituents, mutually interpenetrating in 3D-space, and emphasizing distinct spatial configurations similar to bioinspired Bouligand, brick-and-mortar, and crossed-lamellar architectures. These architectural designs facilitate efficient stress transmission, distribute damage over a wider area, and halt the propagation of cracks, thereby conferring enhanced ductility and strength compared to composites featuring separate reinforcements. Moreover, these materials exhibit the activation of various extrinsic hardening mechanisms, such as crack twist/deflection and uncracked-ligament bridging. These mechanisms effectively shield the crack-tip from the applied stress, resulting in the formation of “Γ”-shaped rising fracture resistance R-curves.

###  Biodegradable metal in 3D printing

####  Magnesium alloys

The implants used in orthopedic surgeries worldwide are predominantly composed of titanium alloys and stainless steels, which are not biodegradable and demand a second procedure for removal or adjustment. The application of inert biometals may also result in the occurrence of long-term complications. In order to address this issue, the implementation of biodegradable biometals has been deemed necessary, with magnesium alloys emerging as a viable and promising alternative. The incorporation of magnesium alloys in implants frequently eliminates the need for a subsequent operation [127, 128]. Furthermore, their biomechanical compatibility surpasses that of titanium alloys or stainless steel, as they possess a density comparable to human bone, resulting in minimal discomfort [129]. The human body has the ability to absorb magnesium and subsequently release it in the form of magnesium ions. These ions play a crucial role in promoting the proliferation and differentiation of osteoblasts, thereby facilitating the process of bone formation and repair [130]. The use of magnesium alloys has been observed in the context of cardiovascular stents as well as orthopedic medical devices such as bone plates and screws. The inherent flammability of magnesium alloys poses challenges in their treatment through laser powder bed fusion. However, there is a growing demand for additively manufactured components made from magnesium alloys. Several studies have been conducted to examine the impact of the parameters of the selective laser melting (SLM) process and the characteristics of magnesium powder on the densification, metallurgical properties, and microstructural defects of magnesium components produced through SLM [131, 132].

Li et al. [133] utilized a 3D printing technique that operates at low temperatures to produce a magnesium-containing degradable polymer that can be used for bone repair. They integrated magnesium, which has angiogenic and osteogenic properties, consistently into a PLGA/TCP biodegradable porous scaffold. The PLGA/TCP/Mg physical structure can be regulated by 3D printing, which includes the concentration and arranged distribution of magnesium and tricalcium phosphate metal. This makes it possible to create an optimum structure for the production of bone, one that has pores that are linked to one another and that improve tissue development, migration, proliferation, and bone cell-adhesion. Additionally, the microscopic framework may be modified to correspond with the form and size of the injured region in patients, which enables individualized restoration. This opens the door to the possibility of regenerative medicine. This biocompatible, bioactive, and strong-yet-degradable polymer bone repair material contains magnesium and is compatible with cancellous bone. Other desirable characteristics include high biocompatibility and biological activity. It also substantially boosts the body’s natural ability to regenerate and create new blood vessels around the implant. Furthermore, inkjet 3D printing technology was applied to create a newberyite (MgHPO_4_. 3H_2_O) and struvite (MgNH_4_PO_4_. 6H_2_O) matrix. This was accomplished by way of a hydraulic establishing reaction involving binder liquid and Mg_3_(PO_4_)_2_ powder, which consisted of either 20% H_3_PO_4_, 0.5 M (NH_4_)_2_HPO_4_, or 2 M K_2_HPO_4_. The post-hardening newberyite compressive strength grew to 36 MPa, while the post-hardening struvite compressive strength increased to 10 MPa. This indicates an increase in the range of 1.3–2.8 MPa. The compressible force of the post-hardening 3D-printed struvite climbed to 10 MPa. This magnesium phosphate matrix, which can be manufactured via the use of 3D printing technology, is anticipated to be an effective biodegradable bone replacement due to the exceptional properties that it possesses [134].

**Fig. 5:**
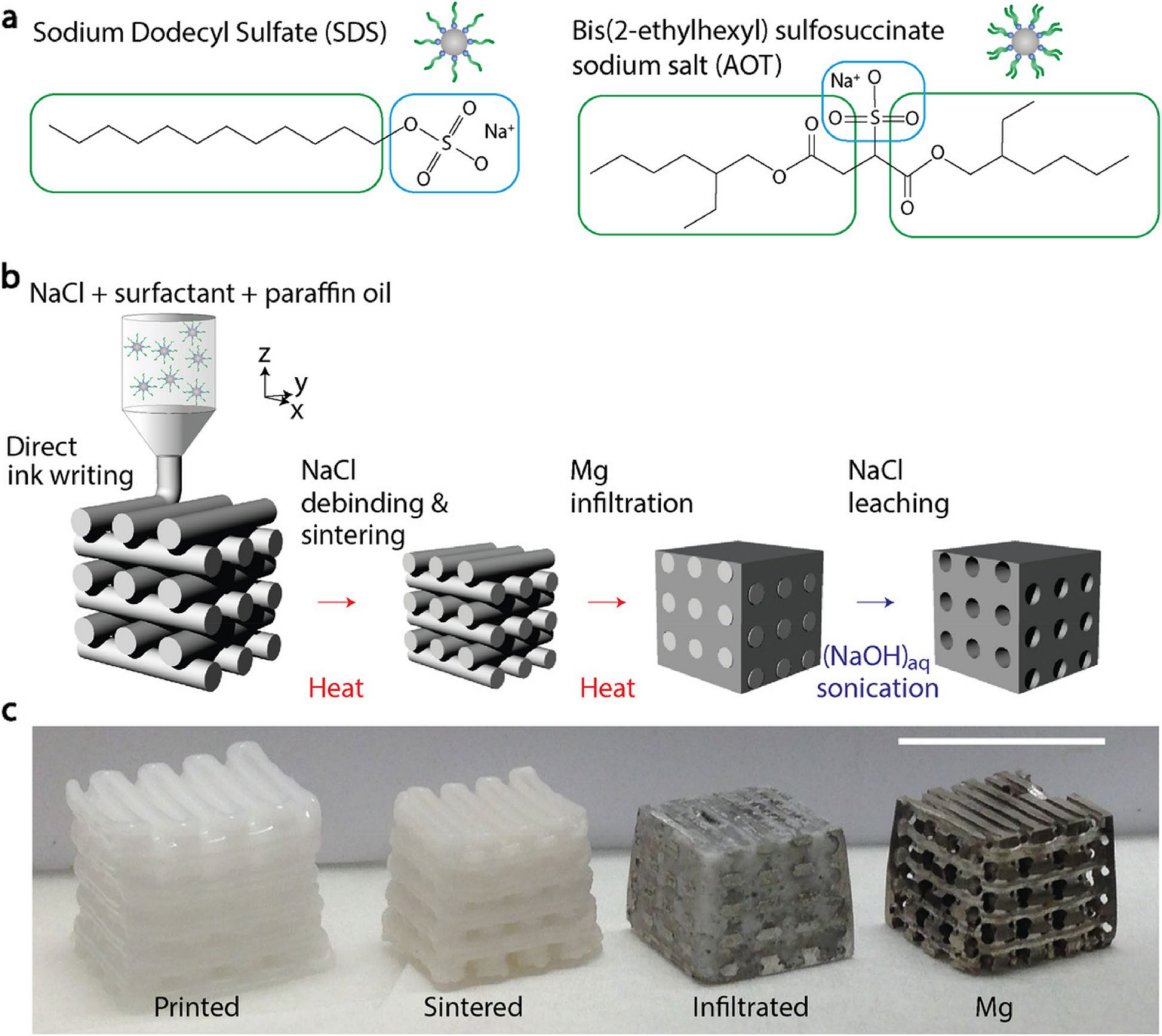
Debinding, sintering, infiltrating, and leaching are the steps involved in 3D printing a NaCl)-based paste onto a porous Mg alloy scaffold. **a** The paste’s chemical composition, including the surfactants SDS and AOT. To facilitate direct ink writing (DIW) printing, sulfonated surfactants are used to fine-tune the interactions among the NaCl granules in the paraffin oil. **b** DIW can 3D print the optimized NaCl paste. In order to infiltrate Mg melt into the printed green body, first the paraffin oil must be removed, and then the body must be calcined and sintered to produce a pure NaCl template. The Mg scaffold takes on the salt template’s structural porosity once the NaCl is leached away. **c** Macrophotographs of finished structures, depicting each stage of the procedure shown in (b) [136]

The use of negative salt pattern molding is a manufacturing method employed to fabricate a magnesium scaffold characterized by an open-cell porous structure, wherein the porosity can be precisely controlled [135]. The use of rapid prototyping and casting techniques facilitates the production of a scaffold that possesses a closely regulated and organized porosity. A high-purity supersaturated NaCl particle paste is prepared, which is then used to produce a 3D-printed positive pattern composed of acrylic polymer. Subsequently, the pattern is infiltrated with a NaCl slurry. The negative salt pattern is impregnated with molten magnesium and dissolved NaCl salt through the use of a NaOH solution. The technique developed by Kleger et al. involved the use of functionalized NaCl particles to produce a paste that exhibits stability, as well as a 3D printable template characterized by structured porosity (Fig. 5) [136]. The salt template undergoes a series of procedures including drying, sintering, and washing with an aqueous solution of NaOH in order to eliminate NaCl following the solidification of molten Mg. The present methodology involves the direct 3D printing of a salt template for the fabrication of a magnesium scaffold, resulting in the formation of a well-organized porous structure. Dong et al. employed the technique of solvent-cast 3D printing to fabricate a porous magnesium scaffold. This was achieved by utilizing metallic powder and binder systems, such as polymer or volatile solvent [137]. The ink paste is expelled at ambient temperature, undergoing a process of solidification through polymerization while maintaining its original form. The compact in question is a metal/polymer composite structure that has been fabricated using 3D printing technology. Through the process of debinding, the composite undergoes a transformation, resulting in the formation of a porous metal structure. The utilization of this technique presents several benefits, including the presence of hierarchical pores, the ability to fabricate at room temperature, the ease of adjusting ink components, and improved control over composition.

The fabrication process employed laser powder bed fusion to synthesize a scaffold made of a porous WE43 alloy, featuring struts of different diameters [138]. The mechanical properties of the short strut scaffold were found to be inferior as a result of shear band propagation. However, the medium strut and long strut scaffolds exhibited notable enhancements in their mechanical properties. Hence, the incorporation of strut design has the potential to greatly improve the mechanical properties of the scaffold. As an example, the LS scaffold exhibited a compressive yield strength of 40 MPa and Young’s modulus of 0.8 GPa. The morphometric parameters and mechanical properties of a WZM211 open porous scaffold were assessed in a study utilizing liquid phase sintering and crucible melt extraction techniques [139]. The scaffold exhibited comparable compressive yield strain to that of natural cancellous bone, though with a lower yield stress. In spite of its reduced mechanical strength, the magnesium alloy scaffold exhibited deformation without the occurrence of microcracks, thus effectively impeding premature failure. The observed decrease in Young’s modulus and hardness following the compression test can be attributed to the formation of Y-Zn-rich precipitates and microdefects, as well as the occurrence of microdamage at the crystallographic level during the process of melt extraction.

Xie et al. fabricated a 3D porous scaffold composed of Mg-Nd-Zn-Zr using the SLM technique, and subsequently conducted an assessment of its compression properties [140]. The compressive yield strength of the 3D printed scaffold was found to be greater in comparison to the soluble template method. A 3D gel printing technique was employed to fabricate a pure magnesium scaffold with three distinct porosities [141]. The compressive strength of the pure magnesium scaffold demonstrated a direct relationship with the sintering temperature, increasing until reaching 610 °C. However, beyond this temperature, the strength of the scaffold decreased, regardless of the wire distance. Additionally, it has been reported that the distance between wires plays a significant role in determining the porosity of the scaffold. The compressive strength of the scaffold experienced a significant decrease as the wire distance was increased. Liu et al. conducted an optimization study on the laser powder bed fusion (L-PBF) technique to fabricate scaffolds using WE43 [142]. The study resulted in the development of four distinct porous units and three different sizes for the struts. The mean hardness value observed was 75 HVN, exhibiting a positive association with the size of the struts. The compressive strength of the scaffolds was found to be the highest among the tested scaffolds. This can be attributed to their distinctive design, which incorporates vertical struts that are parallel to the direction of compression. In contrast, the diamond scaffold exhibited the lowest compressive strength, primarily due to the presence of weak interconnected joints.

####  Iron-based alloys

**Fig. 6:**
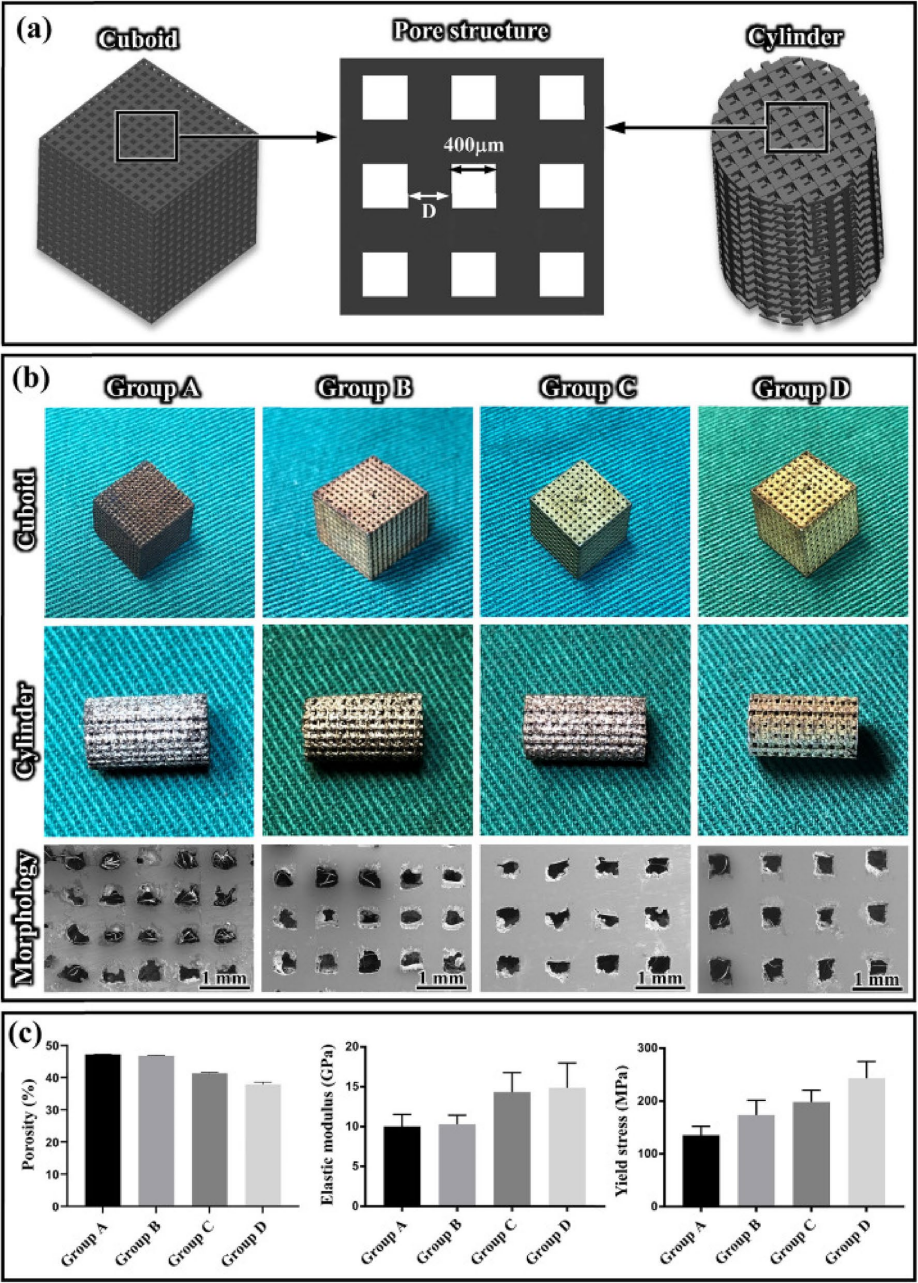
**a** The structural arrangement of porous Fe-30Mn biodegradable scaffolds; (**b**) macro and micro structures of porous Fe-30Mn biodegradable scaffolds (Groups A-D) fabricated via SLM with a cuboid geometry and a cylindrical geometry (base diameter of 6 mm and height of 10 mm); (**c**) yield strength, porosity levels, and elastic modulus values of Groups A-D [147]

Iron is an essential trace element that the human body needs to function properly and is involved in a wide variety of physiological processes. Because of their exceptional mechanical qualities and capacity to degrade, materials based on iron are now being investigated as a potentially fruitful choice for the production of biodegradable implants. Initial in vivo tests on porcine aortas with implanted pure iron stents have shown no local or systemic toxicity, as the iron ions released after degradation can undergo metabolization without accumulation [143]. However, the degradation rate of pure iron in physiological media is slower in comparison to the required body rate. Because of this, researchers have produced a variety of Fe-Mn alloys in an effort to speed up the pace of deterioration of iron-based components[144, 145]. Degradable metals based on iron were some of the first to be employed in 3D-printed scaffolding for use in biomedical applications [146]. Applications in craniofacial reconstruction using inkjet 3D printing of biodegradable Fe-30Mn scaffolds with an average porosity of 36.3% have been investigated (Fig. 6). After further sintering, the scaffold transformed into a mixed phase alloy consisting of martensite and austenite phase. The scaffold’s corrosion rate was significantly higher than that of pure iron, and the result of its corrosion comprised of calcium and phosphorus. The scaffold had outstanding cytocompatibility in addition to possessing tensile mechanical characteristics that were comparable to those of real bone[147].

The manufacturing of porous iron offers a distinct opportunity to enhance the rate of biodegradation through the use of complicated porous architectures. However, obtaining the necessary biodegradation profile keeps presenting challenges as a result of inherent passivation processes and restricted biocompatibility. A coating of poly(2-ethyl-2-oxazoline) was used on porous iron fabricated through extrusion-based 3D printing, resulting in a biodegradation rate that was 2.6 times higher compared to uncoated samples [148]. Furthermore, the mechanical properties of the coated iron remained similar to those of natural bone. The stability of the coating remained intact even after undergoing biodegradation, and it exhibited a significant enhancement in both viability and adherence of preosteoblasts. Putra et al. used extrusion-based 3D printing for the fabrication of iron-matrix composites incorporating akermanite [149]. Iron-based inks, combined with different quantities of akermanite powder, were formulated, followed by meticulous fine-tuning of the debinding and sintering processes. The composite scaffolds effectively maintained their structural integrity and retained the desired α-Fe and akermanite phases in their original form. The in vitro biodegradation rates exhibited an enhancement of 2.6-fold in comparison to pure iron. The scaffolds exhibited enhanced MC3T3-E1 cell attachment and enhanced cell proliferation. The cellular exudation of collagen type-1 and the level of alkaline phosphatase activity exhibited higher values in comparison to Ti6Al4V when subjected to an osteogenic medium. Carluccio et al. reported a detailed examination of Fe-Mg bone scaffolds manufactured using SLM technique, with a specific focus on their suitability for load-bearing applications [150]. The mechanical properties were sufficient for load-bearing applications, with a higher corrosion rate than pure iron due to the manufacturing method, Mn addition, and scaffold design. The results of in vitro cell testing demonstrated favorable viability and biocompatibility, with filopodia exhibiting strong osteoblast adhesion. The in vivo analysis revealed a favorable integration of the bone, as evidenced by the presence of newly formed bone after a period of 4 weeks following implantation.

In addition to iron-based alloys, iron and iron oxide particles have also been investigated for use in 3D-printed scaffolds. Magnetic Fe3O4 nanoparticles, polycaprolactone, and bioactive glass were incorporated into composite scaffolds with the use of a 3D Bioplotter by Zhang et al. [151]. These scaffolds exhibited a compressive strength of 13–16 MPa and a porosity that was evenly distributed throughout the structure of 60%. Due to the presence of magnetic Fe3O4 nanoparticles, the scaffold was endowed with a magneto-thermal effect, which improved the cellular biological capabilities. Additionally, the scaffold was loaded with doxorubicin anticancer drugs to promote osteogenic activity and achieve sustained drug delivery. Artificial magnetic cilium was successfully 3D-printed by Liu et al. [152] using polydimethylsiloxane that had been doped with iron particles. Overall, 3D-printed biodegradable scaffolds made of iron-based materials show promise in promoting bone restoration without problems, making them better candidates for implants of orthopedic. As 3D printing permits the precise fabrication of compound and specific shape implants, it is predicted that degradable iron-based medical devices produced via 3D printing will surpass those produced through conventional techniques.

####  Zinc-based alloys

Zinc (Zn) is a trace element that is vital to the human body and has a key role in the production of enzymes, the expression of genes, the transmission of signals, the metabolism of nucleic acids, the control of apoptosis, the encouragement of growth, and the regeneration of damaged tissue [153]. Researchers have found that Zn has potent anti-atherogenic properties [154]. Despite concerns about the potential adverse effects of using Zn in the body, studies have shown that zinc toxicity is minimal, and it was also found to have antiseptic activity [155]. In recent times, Zn has been emerged as a promising alternative to iron and magnesium alloys in the manufacture of biodegradable medical implants due to its nearly ideal degradation rate. Thus, it has gained attention as a candidate for dental implants besides degradable cardiovascular stents [156].

**Fig. 7:**
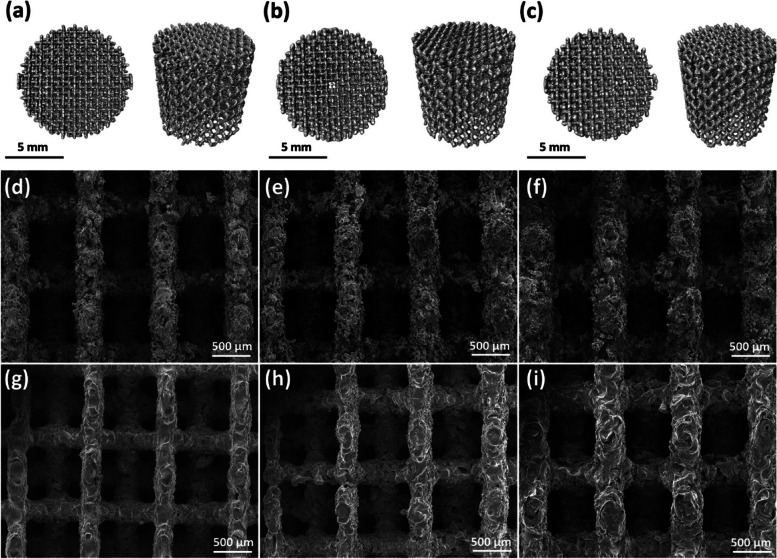
Morphological structures of the porous Zn scaffolds prepared by AM technique [160]

Various studies investigated the fabrication, design, and mechanisms of degradation of Zn alloys in vitro and in vivo [157–159]. However, fabricating Zn implants with SLM is challenging due to its low melting point, low boiling point, and high oxidation tendency resulting in high porosity in fabricated parts. The degree of oxidation level of Zn pieces that were created using a 3D printer was roughly 2 weight%, which is comparable to the oxidation rate of pure Zn powder. Because of the rapid rate of cooling that occurs during the SLM process, the microhardness of Zn pieces that were 3D-printed was somewhat greater than that of pure Zn. By modifying the geometry, density, and microstructure of Zn components that have been 3D-printed, SLM makes it possible to control the mechanical qualities and deterioration of Zn products. Li et al. investigated the characteristics of functionally graded and uniform additive manufacturing (AM) porous Zn designs using a diamond unit cell [160]. Cylindrical specimens were produced using pure zinc powder and subsequently examined to evaluate their parameters and properties. The biodegradation behavior was found to be significantly influenced by the topological design, resulting in a notable 150% variation in biodegradation rates across the three designs. Following 28 days of in vitro biodegradation, the AM porous Zn material exhibited weight losses ranging from 7 to 12%, while concurrently demonstrating a consistent increase in yield strengths (Fig. 7). The topological configuration of porous Zn in additive manufacturing exhibits potential in regulating mechanical characteristics and degradation patterns, thereby providing adaptability to meet diverse clinical demands. Tong et al. reported the development of a biodegradable composite material composed of Zn-Mg-Mg-Si [157]. The composite was fabricated using a high-pressure solidification technique. The composite material exhibited high compression characteristics, with a yield strength of 406.2 MPa and an ultimate strength of 1181.2 MPa. Additionally, it revealed exceptional plastic deformation characteristics, exhibiting no signs of fracturing or cracking. The corrosion potential of the composite material was measured to be -0.930 V. Subsequently, when the composite was immersed in Hanks’ solution, its degradation rate was found to be 42.8 μm/y and 37.8 μm/y. The experimental findings indicate that the extract exhibited favorable cytocompatibility when compared to both pure zinc and AC composites, particularly at concentrations equal to or below 25%. The aforementioned statement implies that the Zn-Mg-Mg-Si composite exhibits considerable potential as a biodegradable material suitable for orthopedic applications. Recently, Qiu et al. examined the pore structure, mechanical properties, and weight loss of porous scaffolds made from Zn-0.8Li alloy in simulated body fluids [159]. NaCl particles were utilized as pore-forming agents. The findings indicated that specimens with smaller pore sizes exhibited superior mechanical properties and reduced rates of degradation. Samples with larger pore sizes exhibited superior connectivity, channels for nutrient transport, and internal tissue growth. The scaffolds composed of Zn-0.8Li, which were coated with chitosan, exhibited enhanced cell activity and adhesion. The application of a chitosan coating resulted in the suppression of Zn^2+^ release, thereby facilitating the growth of bone tissue and creating a favorable microenvironment for the adhesion, proliferation, and differentiation of mesenchymal stem cells.

## Emerging trends and areas of focus

Because it is now possible to make implants that are tailored to the specific requirements of each individual, 3D printing has emerged in recent years as an important technology in the area of medicine. There is a significant increase in the need for customized medicine and custom-made 3D-printed medical products, notably orthopedic instruments and surgical implants, as a result of the fast-growing population of older people around the globe. As a direct consequence of this development, the market for medical devices now prioritizes 3D-printed biodegradable metallic implants. The 3D printing technology provides a number of benefits that have not been seen before, such as the capability to generate specific designs, build structures with complicated geometries, and minimize the amount of time and money spent on the production of biodegradable metallic medical items. Despite this, the implementation of this method in the biomedical field is still in its infancy, and there are a number of obstacles that need to be conquered prior to its clinical application may become widespread.

At the moment, the 3D printing processes for biodegradable composites have not yet been equally well-known as those for traditional manufacturing methods. Additionally, there are a number of significant technological problems and stability problems that need to be solved. These include decreasing the surface roughness, regulating residual stress, improving the relationship between the electron beam and metal powders, eliminating internal structural flaws, and enhancing accuracy and efficiency. Because of variables such as the characteristics of the material, the capabilities of the processing, and the capabilities of the equipment. The layer-by-layer printing process can also lead to product defects and poor performance. In order to implement the 3D printing technology on a broad scale in the medical device industry, it is imperative that these hurdles be surmounted and that high precision, great surface polish, robust mechanical and physical product qualities, and high production speeds be achieved.

Furthermore, there remains a need for higher quality raw materials in 3D printing. Due to the distinct principles of molding used in 3D printing, current raw materials remain often in the form of powders or filaments and must meet specific requirements, such as size, distribution, uniformity, oxygen content, and fluidity, which are more stringent than those for solid materials. Additionally, these materials must meet biological standards to avoid biological risks in medical device applications. However, the number of established materials available for 3D metal printing is very limited. In addition, there are concerns with the equipment that are being used in the present research and development of 3D printing as a method for metal fabrication. These equipment-related issues include expensive purchasing costs, maintenance expenditures, and consumables prices, as well as incompatibility between printing software. These factors have limited the widespread adoption and 3D printing technique development. In order to advance this technology, it is necessary to concentrate on enhancing the high capacity, high throughput, and high resolution of 3D printing equipment in order to achieve miniaturization and cost reduction. In addition to this, it is necessary to improve the compatibility of software and hardware in order to make it easier to implement any future expansions or advancements.

It is imperative that immediate attention be paid to the problem of ensuring that medical items that are manufactured using a 3D metal printer are safe to use. Materials used in clinical settings must meet degradation, stringent safety, biological activity requirements, and biocompatibility to ensure that they are suitable for industrial and clinical use. For instance, the printing process and performance of titanium alloy medical implants must be evaluated to make sure that there are no effects of teratogenicity or carcinogenicity after 3D implantation. However, there is currently no internationally recognized evaluation system for these issues, and there are still ethical concerns to address. There is a dearth of comprehensive understanding regarding the possible dangers associated with the use of 3D-printed implants in clinical settings, despite the fact that there is an increasing demand in the application of 3D-printed prosthetics in clinical settings. It is vital to keep track of the long-term consequences of placing 3D-printed metal devices and to set up an assessment system in order to improve the safety and efficacy of customized medical devices.

## Conclusion

In the present era, 3D printing has gained significant recognition as a groundbreaking technology in the field of customized healthcare owing to its remarkable capacity to fabricate tailor-made orthopedic implants. Ever since the advent of the 3D printing methodology, it has exerted a significant influence on the subject of medical implants. Through the integration of medical imaging modalities with additive manufacturing methodologies, the utilization of additively manufactured implants in the field of orthopedic applications has been significantly expanded. Through the collaborative efforts of surgical professionals and chemical engineers, numerous instances of clinical applications involving the use of 3D-printed implants have been published in the past few decades. The preliminary initiatives have certainly demonstrated the efficacy of employing 3D printing in orthopedic applications. Indeed, 3D printing stands out as a promising methodology capable of overcoming certain complicated clinical challenges. With an increase in proficient researchers entering the discipline of 3D printing, coupled with the constant progress in hardware, software, imaging, and regulation, it is plausible that the enhancements in 3D printed implants will witness swift development and ultimately attain widespread commercial viability in the forthcoming years.

## Data Availability

All the data regarding this manuscript is available in the main text.
